# A Voice Conversion System from Electrolarynx Speech to Preoperative Patient's Speech for Total Laryngectomy

**DOI:** 10.1002/oto2.70207

**Published:** 2026-02-16

**Authors:** Naoki Nishio, Kazuhiro Kobayashi, Ding Ma, Sohei Mitani, Michihiko Sone, Tomoki Toda

**Affiliations:** ^1^ Department of Otorhinolaryngology Nagoya University Graduate School of Medicine 65, Tsurumai‐cho, Showa‐ku Nagoya 466‐8550 Aichi Japan; ^2^ Information Technology Center/Graduate School of Informatics Nagoya University Furo‐cho, Chikusa‐ku Nagoya 464‐8601 Aichi Japan; ^3^ Headquarters TARVO, Inc. Sakae5‐26‐39, Naka‐ku Nagoya 460‐0008 Aichi Japan; ^4^ Department of Otolaryngology‐Head and Neck Surgery Ehime University Graduate School of Medicine Shitsukawa, Toon 791−0295 Ehime Japan

**Keywords:** electrolarynx, laryngectomy, save the voice project, smart device application, voice conversion system

## Abstract

Laryngectomy is a life‐changing procedure typically performed as a surgical treatment for patients with head and neck cancer. Voice conversion (VC) is a technology that converts one voice into another without changing the linguistic information. Recently, “Save the Voice Project” for patients planning to undergo laryngectomy using our VC technique has been initiated. This study evaluated the speech converted from EL speech using the VC technique in a patient with head and neck cancer who had undergone laryngectomy. Additionally, details of an iPhone/iPad application for voice recording were reported. VC technology would greatly improve the quality of life of patients planning to undergo laryngectomy, especially those with relatively well‐preserved vocal function. To preserve the patient's original normal speech, medical staff should perform voice management before and after laryngectomy, and high‐quality voice recordings are essential.

Laryngectomy is a life‐changing procedure typically performed as a surgical treatment for patients with head and neck cancer. Currently, it is estimated that there are approximately 50,000 to 60,000 laryngectomees in the United States. Many studies have indicated the efficacy of surgical voice restoration in improving the quality of life (QoL) among patients after total laryngectomy, such as tracheoesophageal speech, electrolarynx (EL) speech and esophageal speech.^1^, ^2^, ^3^ However, tracheoesophageal speech requires training and speech‐language pathology therapy and is not effective for all patients, highlighting the need for a new, more reliable approach. Ultimately, these alternative speeches are quite different from normal human voices, and the patient's original voice cannot return after total laryngectomy.

Voice conversion (VC) is a technology that converts one voice into another without changing the linguistic information. VC is a process in which the unique characteristics of a speaker's identity are seamlessly transferred to another speaker while preserving the original speech content. This is achieved using algorithms that combine various speech processing techniques, including speech analysis, acoustic feature mapping, and vocoding. Previously, speaking aid systems using neural‐network‐based VC for EL speech were developed, wherein the EL speech of a laryngectomized patient was converted to normal sample speech.^4^, ^5^ Using a statistical VC technique, EL speech could be converted to the patient's original normal speech. This study evaluated the speech converted from EL speech using the VC technique in a patient with head and neck cancer who had undergone laryngectomy. Additionally, details of an iPhone/iPad application for voice recording were reported.

## Methods

### Voice Recording Before and after Laryngectomy

**Figure 1:**
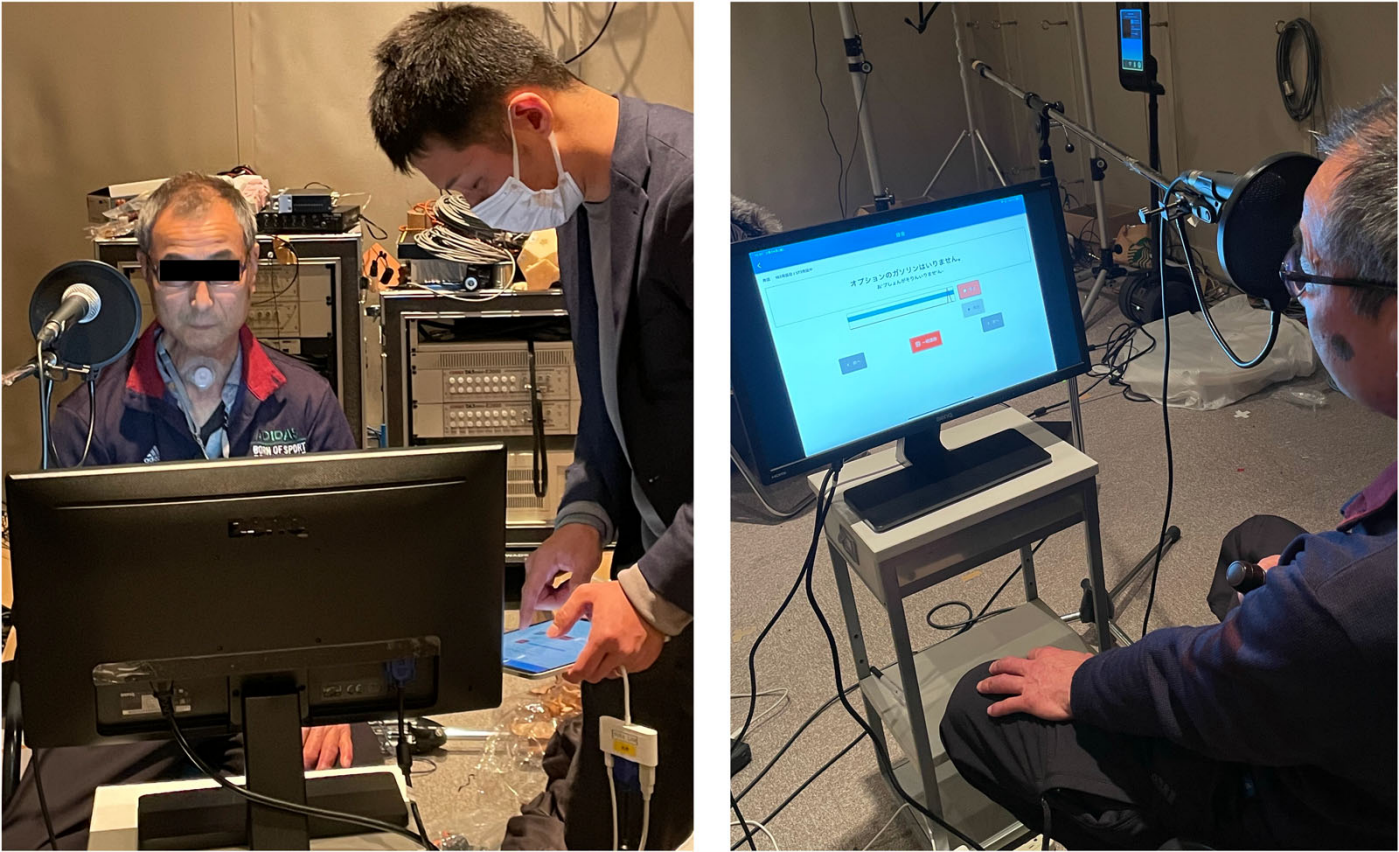
Voice recording after laryngectomy in the studio.

A 63‐year‐old male presented with a 3‐month history of swallowing disorders and left neck swelling. Flexible endoscopic examination revealed a tumor in the hypopharynx, and biopsy revealed squamous cell carcinoma. The patient was diagnosed with hypopharyngeal cancer (cT4aN2bM0) and was scheduled for total pharyngolaryngectomy and free jejunal transfer. Prior to surgery, he spoke 370 sentences and his original voice was recorded in the database. After 6 months postoperatively, his EL speech was recorded via the similar 370 sentences in the studio, as shown in Figure 1. This study was approved by the Ethics Review Committee of Nagoya University Hospital (approval number 2021‐0232). Written informed consent was obtained from the patient.

### Save the Voice Recording Application

**Figure 2:**
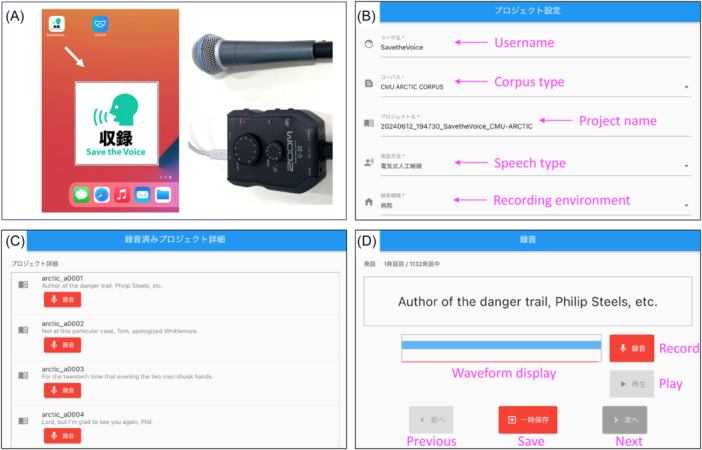
“Save the Voice Recording Application” for iPhone/iPad. (A) Application for iPhone/iPad and recording tools. (B) Project setting. (C) Lists of recording sentences. (D) Recording page.

A voice recording application was developed to save the patient's voice in a database containing several corpora for normal, EL, and shunt speeches. Each corpus contained 200‐1000 sentences to effectively collect voice data. Our recording application, named as “Save the Voice Recording Application,” is described in detail in Figure 2. The application is free and available via the Apple Store and Android Operating System. The recording system was made portable by implementing a recording app for iPhone/iPad. This permits patients to record not only in designated recording rooms but also at any time and place, such as at home, hospital rooms, or examination rooms.

### Neural Network‐Based VC and Perceptual Evaluation

A neural‐network‐based VC method for enhancing EL speech and a novel two‐stage training method that combines a large amount of synthetic parallel data (SPD) generated by text‐to‐speech (TTS) models with small‐scale original parallel data are proposed, as shown in Supplemental Figure S1, available online. Our method utilizes a sequence‐to‐sequence (seq. 2seq) architecture with an attention mechanism to convert EL speech into more natural speech. Of the 370 phoneme‐balanced sentences, 160 sentences and 40 sentences served as training and converted data, respectively.

For perceptual evaluation, 3 subjective metrics were utilized: naturalness (how natural the converted speech sounds), intelligibility (how clearly the converted speech conveyed the intended message), and speaker similarity (similarity of the converted speech with respect to the target speaker). Fifteen native Japanese volunteers, all members of a research lab specializing in VC technology, evaluated patient's “original EL,” “original normal,” and “converted” speeches. Furthermore, “normal sample” from 10 healthy speakers and their corresponding “EL sample” were evaluated. Volunteers were asked to rate the speech samples on an opinion score scale from 1 (Bad) to 5 (Excellent) for naturalness, intelligibility, and speaker similarity.

## Results

**Figure 3:**
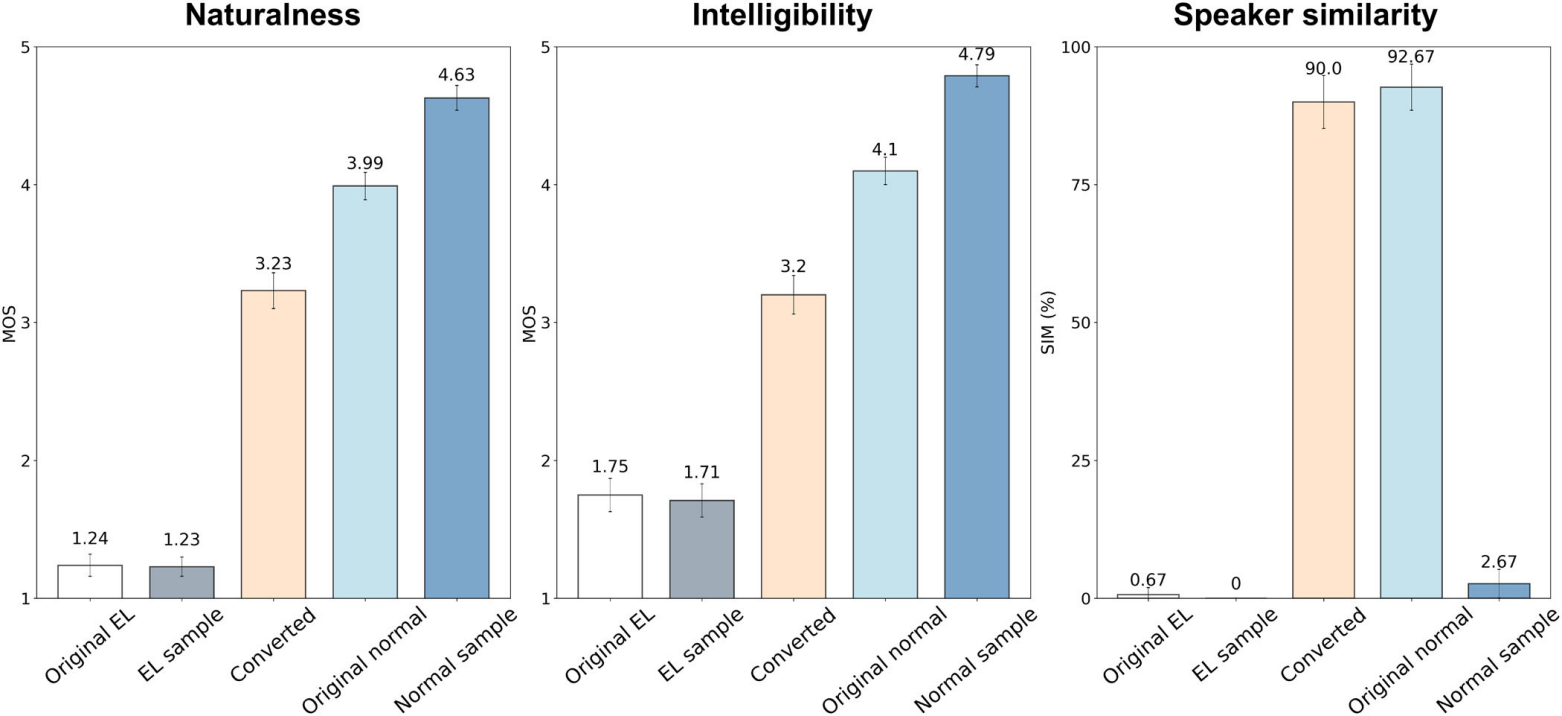
Mean Opinion Score (MOS) for naturalness, intelligibility, and speaker similarity (SIM).

The mean opinion score (MOS) and 95% confidence interval for each speech are described in Figure 3. The mean score for the naturalness of the converted speech (3.2) was much better than that of the patient's EL speech (1.2) or EL sample speech (1.2). Furthermore, the mean intelligibility score of the converted speech (3.2) was much better than that of the patient's EL speech (1.8) and EL sample speech (1.7). These demonstrate that our system dramatically improves the naturalness and intelligibility of a patient's EL speech. The speaker similarity for each speech is presented in Figure 3. Interestingly, the speaker similarity of the converted speech (90.0) was much better than that of the patient's EL speech (0.67), EL sample speech (0), and normal sample speech (2.7), and was quite similar to the speaker similarity of the patient's original normal speech (92.7).

## Discussion

VC systems have primarily been developed for voice disorders such as hoarseness, dysarthria, and neuromuscular diseases in clinical settings. However, only a few studies have focused on the clinical application of VC techniques for EL speech in laryngectomized patients. This study is the first to report a novel neural network‐based VC for EL speech in a patient with head and neck cancer, successfully regenerating the patient's original normal speech using preoperative voice recordings. It is essential to record the original voices in high‐quality settings preoperatively to regenerate them. Clinicians should recognize the significance of preoperative recording of the original voice and manage social and psychological problems for patients and their families to reduce anxiety related to voice loss.

Currently, the “Save the Voice Converting application” has been developed to convert EL speech to the patient's original normal speech for iPhone/iPad or Android devices (as shown in Supplemental Video S1, available online). This converting application enables to convert EL speech to the original speech using a neural network–based VC system. It outputs the converted speech with the same sentences on a smart device within a few seconds after receiving the EL speech input. Previously, objective evaluations were conducted using the character error rate (CER) to assess intelligibility, achieving a best CER of 14.7%, indicating that all EL speech samples were recognizable. These results demonstrate the effectiveness and robustness of our VC system.^4^ Technology in text‐to‐speech systems has already been implemented. In this study, VC technology was applied to patients scheduled to undergo total laryngectomy. This technology can also be used for patients who have already lost their voice. Although perfectly replicating a patient's original voice is challenging, it is feasible to use speech synthesized by text‐to‐speech systems as the target voice.

However, this voice converting application is still under investigation, and several technical issues remain. First, the quality of the converted speech heavily depends on the quality of the recorded EL speech, including its naturalness and intelligibility. The poorer the quality of the input EL speech, the more challenging the conversion process becomes. Furthermore, as the conversion process becomes more difficult, the likelihood of errors during conversion increases. Since a higher‐quality EL speech dataset leads to a more effective VC algorithm, adequate and consistent training for EL speech is essential following total laryngectomy. Second, to regenerate the patient's original speech using VC technology, it is essential to obtain a high‐quality voice recording prior to total laryngectomy. This approach is particularly beneficial for patients whose vocal function is relatively well preserved, such as those with hypopharyngeal cancer or cervical esophageal cancer, but it is not suitable for patients with severely impaired voice or those requiring a tracheostomy. Third, although the converting application produces the converted voice within a few seconds, there is still a slight delay in the conversion process. When patients want to engage in verbal communication, this delay becomes critical, and a real‐time VC system should be developed. Moreover, 2 different speech outputs were generated from the EL device and the smart device in our VC system. Therefore, further research is needed to develop an EL device with an embedded processor capable of accessing the subject's speech database in real time. This application also allows the conversion of EL or shunt speech into the patient's original normal speech in a clinical setting. In the near future, VC system and “Save the Voice” applications allow patients to regenerate their original speech from EL speech, facilitating enjoyable communication with other people in a social community and hopefully resulting in a better QoL before and after laryngectomy. To achieve this, a prospective large‐cohort study is necessary to assess the device's applicability and QoL outcomes in patients at risk of voice impairment—not only those with head and neck cancer but also those with neuromuscular diseases.

## Conclusion

VC technology would greatly improve the QoL of patients planning to undergo laryngectomy, especially those with relatively well‐preserved vocal function. To preserve the patient's original normal speech, medical staff should perform voice management before and after laryngectomy, and high‐quality voice recordings are essential.

## Author Contributions

**Naoki Nishio**, concept/design, analysis, interpretation of data, drafting, and revision; **Kazuhiro Kobayashi**, concept/design, analysis, interpretation of data, drafting, and revision; **Ding Ma**, analysis, interpretation of data, and revision; **Sohei Mitani**, analysis, interpretation of data, and revision; **Michihiko Sone**, analysis, interpretation of data, revision, and supervision; **Tomoki Toda**, concept/design, analysis, interpretation of data, revision, and supervision.

## Disclosures

### Competing interests

None.

### Funding source

This study was partially supported by JST CREST JPMJCR19A3, AMED under Grant Number JP21dk0310114, and JSPS KAKENHI Grant Number JP25K02788.

## Supporting information

Supplemental Figure 1. Synthetic parallel data (SPD) generation and two‐stage electrolarynx (EL)‐speech‐to‐normal‐speech (EL2SP) training.

**Supplemental Video 1.** Save the Voice Project, A Voice Conversion System for Total Laryngectomy.

## Data Availability

All data that support the findings of this study are available from the corresponding author upon reasonable request.
