# Supplementary figures and images for: A Voice Conversion System from Electrolarynx Speech to Preoperative Patient's Speech for Total Laryngectomy
Source: OTO Open. 2026 Feb 16;10(1):e70207. doi: 10.1002/oto2.70207 (PMC12908420; doi:10.1002/oto2.70207)

## Extend small-scale EL2SP dataset with SPD

- Data preparation

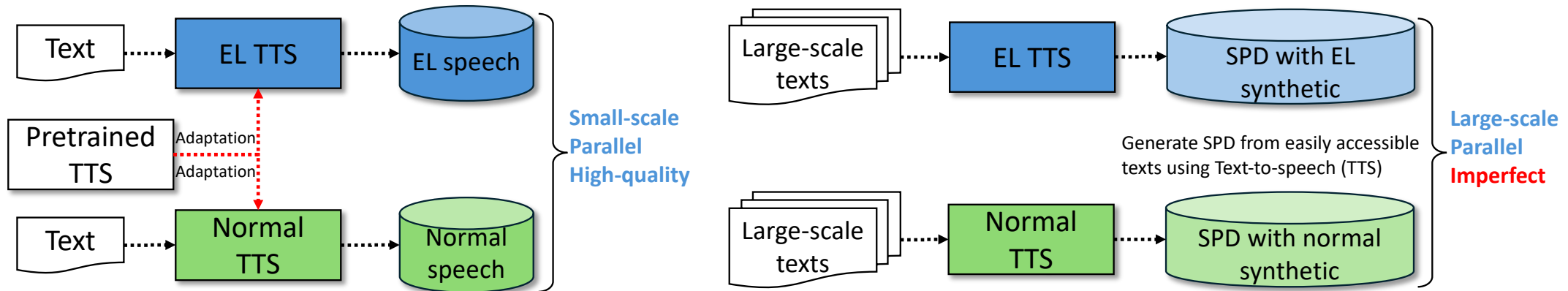

- EL2SP model training

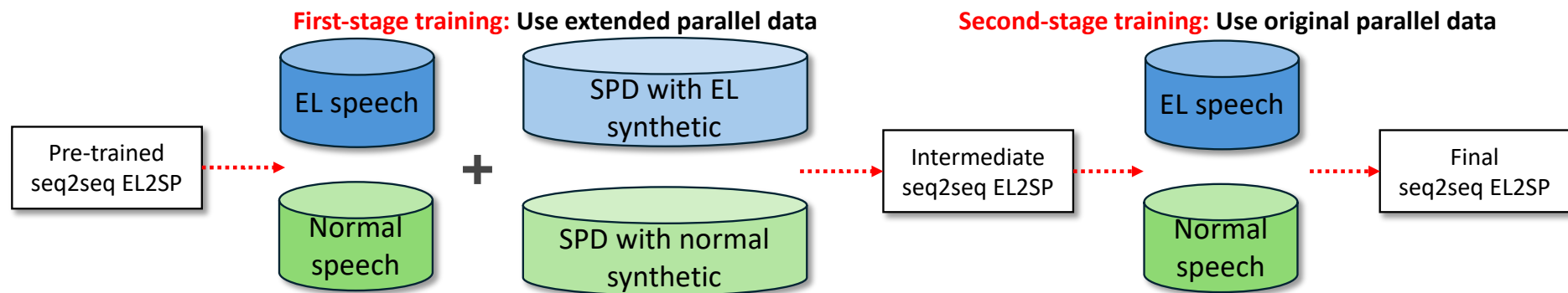

Supplement: Supplementary file 1 — Supplemental Figure 1. Synthetic parallel data (SPD) generation and two‐stage electrolarynx (EL)‐speech‐to‐normal‐speech (EL2SP) training. [file OTO2-10-e70207-s001.pdf]
